# Identification of a ZC3H12D-regulated competing endogenous RNA network for prognosis of lung adenocarcinoma at single-cell level

**DOI:** 10.1186/s12885-021-08992-1

**Published:** 2022-01-28

**Authors:** Wenhan Chen, Zhifeng Guo, Jingyang Wu, Guofu Lin, Shaohua Chen, Qinhui Lin, Jiansheng Yang, Yuan Xu, Yiming Zeng

**Affiliations:** 1 Department of Respiratory Pulmonary and Critical Care Medicine, The Second Affiliated Hospital of Fujian Medical University, Quanzhou, 362000 Fujian Province China; 2 Respiratory Medicine Center of Fujian Province, Quanzhou, 362000 Fujian Province China; 3 The Second Clinical College, Fujian Medical University, Fuzhou, 350004 Fujian Province China; 4 Department of Thoracic Surgery, The Second Affiliated Hospital of Fujian Medical University, Quanzhou, 362000 Fujian Province China; 5 Department of Pathology, The Second Affiliated Hospital of Fujian Medical University, Quanzhou, 362000 Fujian Province China

**Keywords:** Zinc finger CCCH-type containing 12D (ZC3H12D), Competitive endogenous RNA, Single-cell RNA sequencing, Lung adenocarcinoma, Immunomics

## Abstract

**Background:**

To identify hub genes from the competing endogenous RNA (ceRNA) network of lung adenocarcinoma (LUAD) and to explore their potential functions on prognosis of patients from a single-cell perspective.

**Methods:**

We performed RNA-sequencing of LUAD to construct ceRNA regulatory network, integrating with public databases to identify the vital pathways related to patients’ prognosis and to reveal the expression level of hub genes under different conditions, the functional enrichment of co-expressed genes and their potential immune-related mechanisms.

**Results:**

ZC3H12D-hsa-miR-4443-ENST00000630242 axis was found to be related with LUAD. Lower ZC3H12D expression was significantly associated with shorter overall survival (OS) of patients (HR = 2.007, *P* < 0.05), and its expression was higher in early-stage patients, including T1 (*P* < 0.05) and N0 (*P* < 0.05). Additionally, ZC3H12D expression was higher in immune cells displayed by single-cell RNA-sequencing data, especially in Treg cells of lung cancer and CD8 T cells, B cells and CD4 T cells of LUAD. The functional enrichment analysis showed that the co-expressed genes mainly played a role in lymphocyte activation and cytokine-cytokine receptor interaction. In addition, ZC3H12D was associated with multiple immune cells and immune molecules, including immune checkpoints CTLA4, CD96 and TIGIT.

**Conclusion:**

ZC3H12D-hsa-miR-4443-ENST00000630242 ceRNA network was identified in LUAD. ZC3H12D could affect prognosis of patients by regulating mRNA, miRNA, lncRNA, immune cells and immune molecules. Therefore, it may serve as a vital predictive marker and could be regarded as a potential therapeutic target for LUAD in the future.

**Supplementary Information:**

The online version contains supplementary material available at 10.1186/s12885-021-08992-1.

## Background

Lung adenocarcinoma (LUAD), accounting for approximately 60% of non-small cell lung cancer (NSCLC), is the most common subtype of lung cancer with a high incidence worldwide [1, 2]. With the advancement of technological innovation, the mechanisms behind LUAD are gradually revealed. Growing evidence highlights the vital role of the competitive endogenous RNA (ceRNA) regulatory networks in LUAD. For instance, AC079160.1-miR-539-5p-CENPF axis may participate in hypoxia-induced tumor cell stemness of LUAD. Low expression of AC079160.1 and CENPF and high expression of miR-539-5p were correlated with hypoxia and stemness index, indicating a better prognosis [3]. Furthermore, linc01833-miR-519e-3p-S100A4 axis might be associated with LUAD progression, and linc01833 overexpression can significantly improve proliferation and invasion ability of lung cancer cells as well as promote the epithelial-mesenchymal transformation process [4].

Although the function of ceRNA network concerning LUAD has been uncovered gradually, the role of single-cell RNA sequencing (scRNA-seq) in LUAD is still waiting to be explored. ScRNA-seq is a novel hot spot to demonstrate the underlying mechanisms of LUAD, uncovering new differentially expressed genes as well as the heterogeneity of immune response-related genes [5, 6]. Besides, scRNA-seq was applied to unravel the molecular and cellular reprogramming mechanisms in metastatic LUAD and the cell-cycle state of the circulating tumor cells of cerebrospinal fluid in LUAD patients with leptomeningeal metastases [7, 8]. When it comes to immunotherapy, scRNA-seq technology might locate the expression of hub genes in immune cells of LUAD to improve precision of immunotherapy in the future [9]. Therefore, integrating ceRNA network with scRNA-seq data may provide a promising strategy for further understanding the underlying mechanisms of LUAD, and might collect more valuable knowledge to improve individual targeted treatment.

At present, there is no investigation on zinc finger CCCH-type containing 12D (ZC3H12D)-hsa-miR-4443-ENST00000630242 axis, which might play a critical role in LUAD. Anti-oncogene ZC3H12D, also called p34, is a member of the zinc finger CCCH-type protein family that is associated with gene expression such as IER3, TNF, IL-6, NF-κB and TLR, degrading inflammatory transcripts and attenuating macrophage response [10–12]. Previous studies have demonstrated that ZC3H12D could be targeted by miR-128-3p, involving in cell proliferation and migration in osteosarcoma [13]. However, the functions of hsa-miR-4443 in different cancers are heterogeneous. It could promote the drug resistance to epirubicin in NSCLC [14]; While its overexpression of hsa-miR-4443 acts in a tumor-suppressive manner, decreas the invasiveness of hepatocellular carcinoma (HCC) [15], glioblastoma (GBM) [16] and colorectal cancer (CRC) [17]. Next, long non-coding RNAs (lncRNAs) have been demonstrated their roles in ceRNA networks as regulators,  participating in the regulation of various pathological processes related to cancers. Compared with the mRNA and miRNA, little is known about the function of lncRNA FAM30A. Highly expressed in B cells, FAM30A is correlated with the regulation of immune response and immunoglobulin genes [18, 19]. Therefore, our study aims to reveal the function of the ZC3H12D-hsa-miR-4443-ENST00000630242 axis in the prognosis of LUAD, especially the role of ZC3H12D concerning immunomics.

## Methods

### Patients and clinical samples

Ten paired LUAD and paracancerous tissues were collected between January 1, 2019 and May 31, 2019 at Fujian Medical University Second Affiliated Hospital. All samples were obtained from LUAD patients who only received primary surgical treatment. Patient characteristics are provided in Table 1. All the Surgically-resected samples were immediately snap-frozen by liquid nitrogen, and then transferred to a − 80 °C refrigerator for RNA extraction. According to the World Health Organization (WHO) guidelines (2015), we had two well-experienced pathologists to confirm the clinicopathological diagnosis. The study was approved by the bioethical committees at The Second Affiliated Hospital of Fujian Medical University, China (2020-206). And, all participating patients provided written informed consent.

**Table 1:** Clinical information of 10 LUAD patients involved in the study

No.	Sex	Histology	Primary site	TNM
RT19004	Male	LUAD	Right upper lung field	P-T2N0M0 Ib
RT19007	Female	LUAD	Right upper lung field	P-T2aN0M0 Ib
RT19008	Male	LUAD	Left upper lung field	P-T1cN0M0 Ia
RT19009	Female	LUAD	Left upper lung field	P-T1aN0M0 Ia
RT19011	Female	LUAD	Right upper lung field	P-T1bN0M0 Ia
RT19012	Female	LUAD	Left lower lung field	P-T1cN0M0 Ia
RT19014	Female	LUAD	Right lower lung field	P-T1bN0M0 Ia
RT19015	Male	LUAD	Right lower lung field	P-T1bN0M0 Ia
RT19018	Male	LUAD	Right upper lung field	P-T1bN0M0 Ia
RT19021	Male	LUAD	Right lower lung field	P-T1bN0M0 Ia

*LUAD* lung adenocarcinoma

### RNA extraction and sequencing

Using the RNeasy Mini Kit (Qiagen, Germany) to isolate the total RNA of LUAD tissues and paracancerous tissues from collected frozen tissues following the standard manufacturer’s instructions. Qubit 4.0 (Thermo Fisher Scientific, Wilmington, DE, USA) was used to evaluate the RNA concentration, and agarose gel electrophoresis was applied to assess the RNA quality.

Then, the maximum residual non-coding RNA (ncRNA) was retained after the ribosomal RNA was removed from the total RNA. Using the TruSeq RNA Sample Prep Kit (Illumina, San Diego, CA, USA) to perform the cDNA library construction after fragments of rRNA-depleted RNA. Following the standard manufacturer’s instructions, the VAHTS total RNA-seq Library Prep kit for Illumina (Vazyme NR603, China) was used for generating lncRNA/mRNA sequencing libraries. The 150-bp paired-end reads’ cDNA fragments were generated for RNA sequencing. Then, establish the miRNA library for samples using the NEBNext® Multiplex Small RNA Library Prep Set for Illumina® (NEB). With a single lane of Illumina HiSeq Xten sequencing platform, 12 libraries were pooled and sequenced. And establish the miRNA library with 50-bp paired-end reads using the Illumina’s TruSeq small RNA library preparation kit. Using the Illumina HiSeq Xten platform to carry out the sequencing for both lncRNA/mRNA and miRNA after library construction.

### Identifications of differentially expressed mRNAs, miRNAs and lncRNAs

Mirdeep2 (v2.0.0.5) was applied to predict new miRNA, whose expression was calculated and standardized using counts per million (CPM) read [20]. While, LncRNAs were annotated by these databases, including CNCI (https://github.com/www-bioinfo-org/CNCI) [21], CPC2 (http://cpc2.cbi.pku.edu.cn/) [22], CPAT (https://sourceforge.net/projects/rna-cpat/) [23] and PLEX (https://sourceforge.net/projects/plek/) [24]. The intersection was retained for further analysis.

Screen the differentially expressed mRNAs (DEmRNAs), differentially expressed miRNAs (DEmiRNAs) and differentially expressed lncRNAs (DElncRNAs) between LUAD and normal tissues using the DESeq2Rpackage (https://bioconductor.org/packages/release/bioc/html/DESeq2.html) in the Bioconductor project. The cut-off criteria was |Log2(fold change)|(|log2FC|) ≥1 and statistical *P* < 0.05. Subsequently, unsupervised hierarchical clustering was performed for DE-RNAs https://cran.r-project.org/web/packages/pheatmap/index.html.

### Analysis of the DElncRNAs enrichment pathway

Using the gene ontology (GO, http://www.bioconductor.org/packages/release/bioc/html/topGO.html) function analysis to screen enrichment of targeted genes to annotate the biological functions regulated by DElncRNAs, including molecular function (MF), biological processe (BP), and cellular component (CC). In addition, using the Kyoto Encyclopedia of Genes and Genomes (KEGG, http://www.genome.jp/keggbin/show_organism?menu_type=pathway_maps&org=hsa) analysis to determine the vital signaling pathways associated with DElncRNAs. Both GO and KEGG enrichment analysis set gene count ≥2 and *P* value < 0.05 as the threshold for statistical significance.

### Predication of miRNA regulation relationship

The miRWalk 2.0 (http://zmf.umm.uni-heidelberg.de/pps/zmf/mirwalk2/) was applied to perform the prediction of miRNA-gene analysis of DEmiRNA [25]. Additionally, using the miRWalk, miRanda, miRDB, miRMap and TargetScan databases to predict the potential DEmiRNA-DEmRNA regulatory relationships. Subsequently, the StarBase (http://starbase.sysu.edu.cn/) database was used to predict the potential miRNA-lncRNA regulatory relationships by DEmiRNAs [26]. Then, based on shared DEmiRNAs with which DEmRNAs and DElncRNAs interact, the DEmRNA-DEmiRNA and DEmiRNA-DElncRNA regulatory relationships were successfully constructed, and were visualized by Cytoscape software.

### Construction of lncRNA-miRNA-mRNA ceRNA network

Based on the hypothesis of miRNA sponge, we focused on the positive correlation expression of DElncRNAs-DEmRNAs, and obtained the co-expressed relationships between DEmRNAs and DElncRNAs simultaneously regulated by DEmiRNAs. Subsequently, based on shared miRNAs, we constructed the competing endogenous RNA (ceRNA) network. Furthermore, we applied a hypergeometric cumulative distribution function test to predict the possible ceRNA pairs, and only the pairs with correlation coefficient>0.5 and *P* value <0.05 were selected. The Sankey diagram was built based on the R software package ‘ggalluval’ (https://github.com/corybrunson/ggalluvial).

### Public data source

Raw counts of RNA-sequencing data and corresponding clinical information were obtained from The Cancer Genome Atlas (TCGA) dataset (https://portal.gdc.cancer.gov/) in January 2020, and the method of acquisition and application for data were complied with the guidelines and policies. LUAD-related RNA-sequencing data and corresponding clinical information were obtained from the TCGA database as following criteria: 1) histologically diagnosed as LUAD; 2) data with clinical information. Finally, a total of 510 paired LUAD tissues was included for further analysis.

MiRNAs data was downloaded from KM-plotter (https://kmplot.com/analysis/) [27], and LncRNAs data was obtained from TCGA database. The single-cell sequencing data of normal lung tissue from 8 mice came from Tabula Muris (https://tabula-muris.ds.czbiohub.org/) [28] https://www.synapse.org/. While, the single-cell sequencing data of NSCLC, including LUAD, resulted from the GEO database (https://www.ncbi.nlm.nih.gov/geo/) and European Molecular Biology Laboratory (EMBL, https://www.ebi.ac.uk/). The databases were included only if they contained single cell RNA sequencing of NSCLC. Finally, we founded 6 valuable datasets. The ScRNA-seq data of EMTAB6149 was prepared from 5 dissected lung tumors [29], while GSE117570 contained scRNA-seq data of tumor-infiltrating immune cells from 4 untreated NSCLC patients [30]. Besides, GSE127465 contained scRNA-seq data of red blood cell (RBC)-depleted cells from NSCLC tumor and from blood of 7 patients, as well as CD45-positive cells from lungs from two healthy mice and two tumor-bearing mice [31]. GSE127471 data were collected from cryopreserved peripheral blood mononuclear cells of NSCLC and GSE131907 contained data of 208,506 cells derived from 44 patients with LUAD, taken from normal lung tissues, LUAD tissues, normal lymph nodes, invaded lymph nodes, pleural effusion, and brain metastases [32, 33]. Additionally, GSE139555 contained data of pretreatment samples from 14 NSCLC patients, which covered normal tissues, primary tumors and peripheral blood [34]. The present study meets the criteria of data usage and publishing of the National Cancer Institute of National Institutes of Health.

### Survival prognosis and clinical factors

The Kaplan–Meier survival analysis was used to compare the survival difference between the above two groups using ‘survival’ (https://cran.r-project.org/web/packages/survival/index.html) and ‘survminer’ (https://cran.r-project.org/web/packages/survminer/index.html) R packages. When processing mRNA or lncRNA expression data, we splited patients by median. However, due to zero miRNA expression data in some LUAD samples, we decided to use auto select best cutoff to group patients better. TimeROC analysis was performed to compare the predictive accuracy of each gene and risk score. For Kaplan–Meier curves, *p*-values and hazard ratio (HR) with 95% confidence interval (CI) were generated by log-rank tests. Univariate and multivariate cox regression analysis were performed to identify the proper terms to build the nomogram. The forest plot was used to display *P* values, HR and 95% CI of each variable using ‘forestplot’ R package (https://CRAN.R-project.org/package=forestplot). Nomograms were developed to predict LUAD patients' overall survival (OS) at 1, 3, 5 years, respectively, based on the results of multivariate Cox proportional hazards analysis. The nomograms quantified the risk so that we could evaluate the prognosis of LUAD patients by the points associated with each risk factor through ‘rms’ R package (https://cran.r-project.org/web/packages/rms/index.html) [35]. Both the above analysis methods and R packages were implemented by R foundation for statistical computing (2020) version 4.0.3 and ggplot2 (v3.3.2). *P* value< 0.05 was considered statistically significant.

### Gene expression atlas based on single-cell RNA sequencing data

We chose the t-distributed Stochastic Neighbor Embedding (t-SNE) algorithm [36, 37] or Uniform Manifold Approximation and Projection for Dimension Reduction (UMAP) [38] to reduce the dimensionality of the quality-controlled scRNA-seq data of normal lung cell from mouse [39]. Moreover, scRNA-seq data from lung cell atlas of human [40] and lung cancer brain metastases [41] of human were re-analyzed through the UCSC cell browser (https://cells.ucsc.edu/) [42]. To understand the expression of ZC3H12D in different cell types across selected datasets, we applied TISCH (http://tisch.comp-genomics.org/) to obtain the ZC3H12D average expression data from multiple databases [43]. Furthermore, we explored the expression of ZC3H12D in LUAD and other well-characterized NSCLC at single-cell level and identified the distribution of expression of ZC3H12D in different crucial cell-types across datasets. The expression of ZC3H12D was collapsed by mean value. The gene expression level displayed using UMAP and violin plots was quantified by the normalized values.

### Screening of co-expressed genes and enrichment analysis

We applied Spearman’s correlation analysis to identify genes that were related to the expression level of ZC3H12D. Those genes with correlation coefficient>0.5, *P* < 0.01 and FDR < 0.01 were considered as co-expressed genes for GO functional enrichment analysis and KEGG pathway enrichment analysis. The top 10 categories by GO analysis and top 10 pathways enriched by KEGG analysis were displayed, respectively. The volcano plot and heat map were drawn by LinkedOmics (http://www.linkedomics.org/login.php) [44] and Metascape (http://metascape.org/) [45], and the bubble diagram was drawn by ‘ggplot2’ R package (https://cran.r-project.org/web/packages/ggplot2/index.html). The sangerbox tool also provided assistance with image adjustments during drawing, a free online data analysis platform (http://www.sangerbox.com/tool).

### The immune cell infiltration level and immune molecules expression

To obtain reliable immune infiltration estimations, we utilized the ‘immunedeconv’(https://www.rdocumentation.org/packages/immunedeconv/versions/2.0.0) R package that integrated six state-of-the-art algorithms [46], including Estimating the Proportion of Immune and Cancer cells (EPIC) [47] and Tumor Immune Estimation Resource (TIMER, http://timer.cistrome.org/) [48]. Besides, immunostimulators and immunoinhibitors were selected and the expression levels (transcripts per kilobase of exonmodel per million mapped reads) of these genes were extracted. The two-gene, and the multi-gene correlation map was displayed using ‘ggstatsplot’ (https://cran.r-project.org/web/packages/ggstatsplot/index.html) and ‘pheatmap’ R package (https://cran.r-project.org/web/packages/pheatmap/index.html), respectively. Additionally, we used Spearman’s correlation analysis to describe the correlations among gene expression levels. And *P* < 0.05 was considered statistically significant.

## Results

### Construction of ceRNA regulatory network in LUAD

**Fig. 1:**
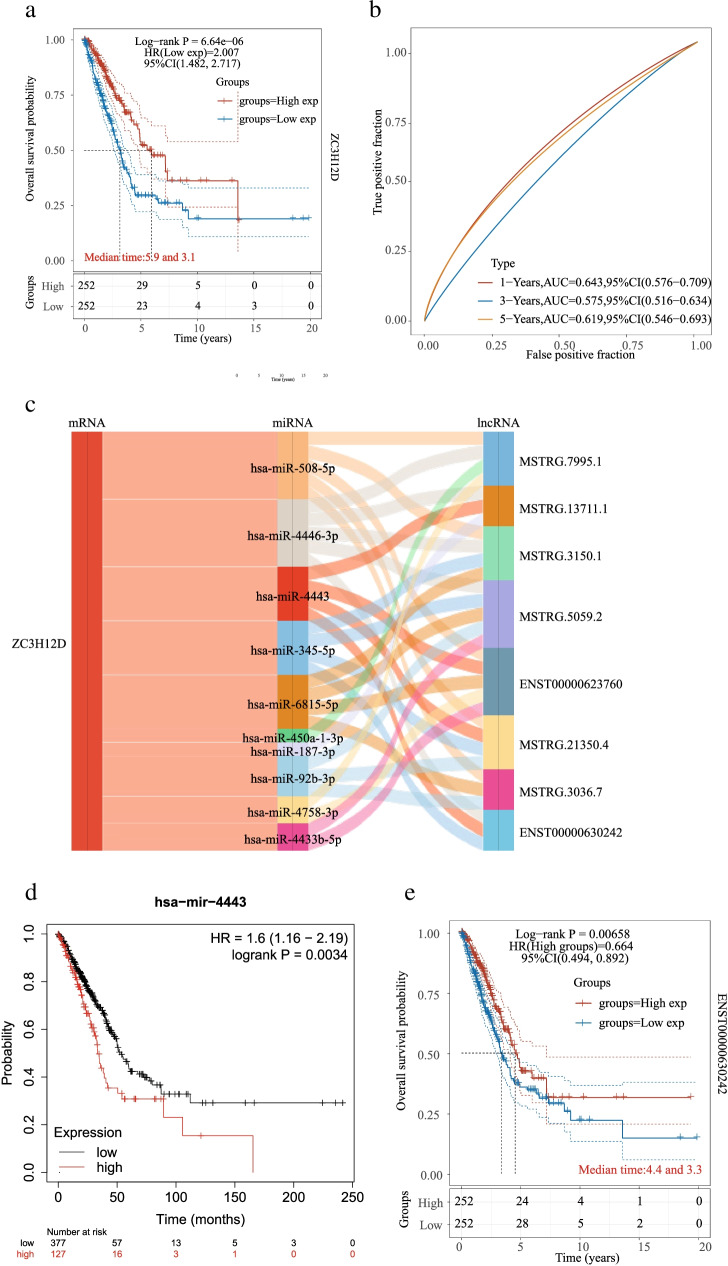
The lncRNA-miRNA-mRNA ceRNA networks is constructed. **a** Survival analysis of ZC3H12D. ZC3H12D was identified to be significant associated with OS. **b** TimeROC analysis of ZC3H12D in LUAD. A higher AUC value indicated a better predictive power for ZC3H12D. **c** The ceRNA network constructed by ZC3H12D and the associated miRNAs and lncRNAs. **d** Survival curve of hsa-miR-4443. High expression of hsa-miR-4443 was not conducive to the prognosis of patients with LUAD. **e** Survival curve of ENST00000630242 (FAM30A). High expression of ENST00000630242 was beneficial to the prognosis of patients with LUAD

Pearson correlation coefficient and significant *p* value between mRNAs and lncRNAs expression were calculated for differentially expressed miRNAs and their differentially expressed target mRNAs and lncRNAs, and for all known miRNAs and their differentially expressed target mRNAs and lncRNAs. In the present study, a total of 1955 differentially expressed mRNAs, 165 differentially expressed miRNAs and 1107 differentially expressed lncRNAs were identified by the RNA sequencing profiles of 10 stage I LUAD patients. The top 50 miRNAs and their target genes were ranked according to the respective network degree of both mRNAs as well as lncRNAs for display. Totally, 49 mRNAs, 99 miRNAs and 50 lncRNA were obtained to construct ceRNA regulatory networks (Supplement 1a). To identify a powerful pathway from this complicated ceRNA regulatory network, we focused on the function of lncRNA-miRNA-mRNA axises that could predict OS of LUAD. Verified by the Kaplan–Meier survival analysis, five mRNAs including ZC3H12D were identified to be significant associated with OS, including ZC3H12D, GNAO1, KSR2, SBK1 and SLIT3 (Fig. 1a-b and Supplement 2). Compared with TCGA normal data, LUAD samples had significantly higher expression of SBK1 and ZC3H12D, while lower SLIT3 expression was observed in LUAD. However, there was no significant difference in the expression of GNAO1 and KSR2 between tumor and paracancerous tissue samples. Meanwhile, both SBK1 and SLIT3 were found to play a vital role in lung cancer by miRNA or lncRNA [49–51]. Thereinto, we focused on ZC3H12D to construct a ceRNA regulatory network, and screened for potentially functional miRNAs and lncRNAs (Fig. 1c). Then, high expression of hsa-miR-4443 was not conducive to the prognosis of patients with LUAD (Fig. 1d), but high expression of ENST00000630242 was beneficial to the prognosis of patients with LUAD (Fig. 1e). Furthermore, ZC3H12D-hsa-miR-4443-ENST00000630242 axis was established, which might play a crucial role in the prognosis of LUAD patients.

### Establishment of cox prognostic model

**Fig. 2:**
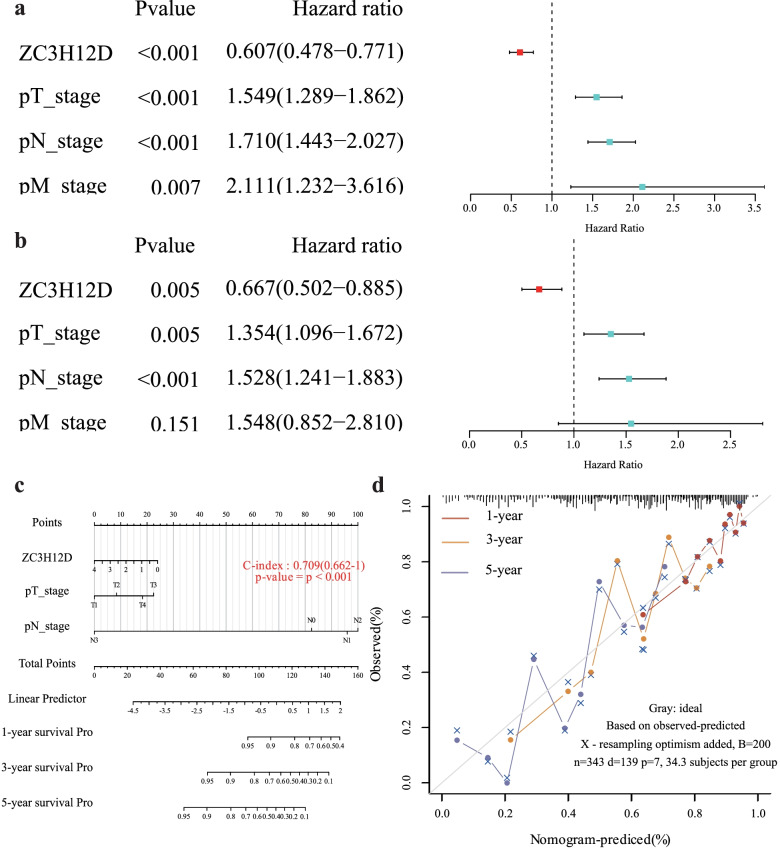
The Cox regression models. ZC3H12D was beneficial to the overall survival of patients in both **a** univariate and **b** multivariate Cox regression models. **c** Nomogram to predict the 1-y, 3-y and 5-y overall survival of LUAD patients. A nomogram was built based on multivariate Cox proportional hazards analysis to predict the X-year overall survival, calculating the risk by the points associated with each factor. A higher score indicated a higher risk of death. Calibration curve for the OS nomogram model of ZC3H12D. **d** A red line represented the 1-y observed nomograms, an orange line represented the 3-y observed nomograms, **a** blue line represented the 5-y observed nomograms and a gray diagonal line represents the ideal nomogram. The nomogram predicting 1-year OS was relatively more accurate than the nomogram predicting 3-year OS or 5-year OS of LUAD patients

To explore the predictive role of ZC3H12D in LUAD patients, we constructed Cox regression models. Fortunately, ZC3H12D was beneficial to the overall survival of patients in both univariate and multivariate Cox regression models. (Fig. 2a and b). Based on the identifications from the multivariate cox proportional hazards analysis, a prediction nomogram was developed to calculate the risk by the points associated with the three risk factors (ZC3H12D, N stage, T stage). The total score ranged from 0 to 160, and was calculated by summing all the scores in each variable. According to the score calculated, we could roughly predict one-year survival rate, three-year survival rate and five-year survival rate of patients. A higher score indicated a higher risk of death (Fig. 2c). For the purpose of internal validation, the calibration plot showed that the nomogram predicting 1-year OS was relatively more accurate than the nomogram predicting 3-year OS and 5-year OS of LUAD patients (Fig. 2d).

### Relationship between vital clinical factors and ZC3H12D expression level

**Fig. 3:**
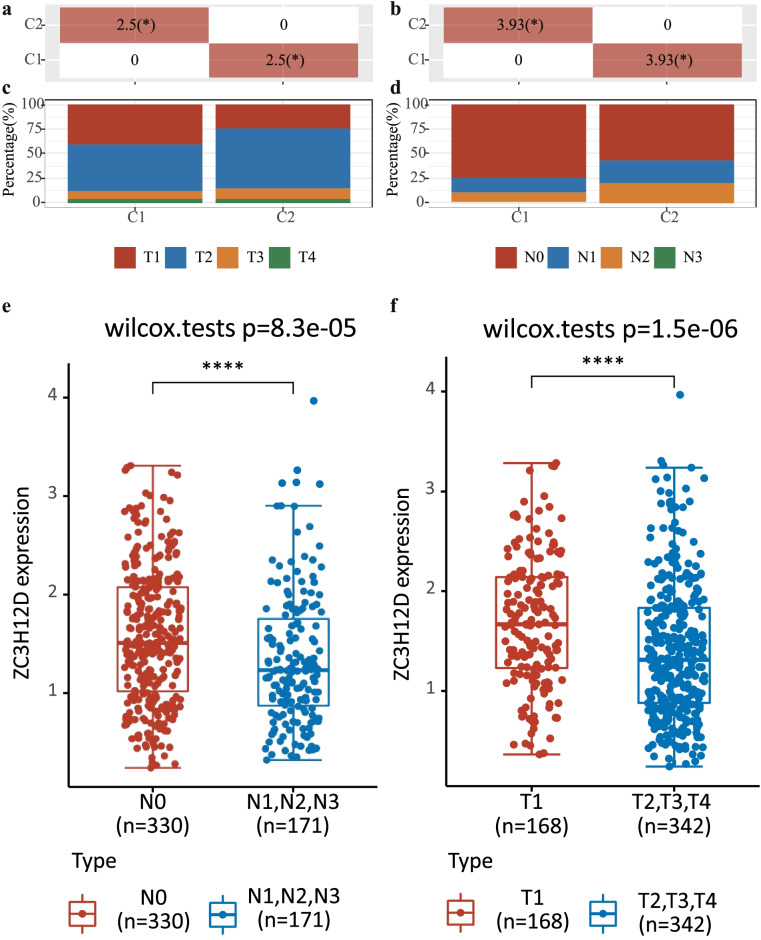
The relationship between ZC3H12D expression and clinical factors associated with prognosis. In group C1, the expression of ZC3H12D was higher than the median, while in group C2 it was lower than the median. **a** The chi-square test for the distribution of tumor size. **b** The chi-square test for the distribution of metastasis to lymph nodes. The correspondent bar chart exhibited the proportion of ZC3H12D high expression in **c**) T1 and **d**) N0 was significantly higher than that of ZC3H12D low expression. **e** Box plots of ZC3H12D expression between N0 group and the lymph node metastasis group. In N0 group, the ZC3H12D expression was significantly higher than another group. **f** Box plots showed that the ZC3H12D expression was relatively higher in T1 phase than that in the T2, T3 and T4. * *p* < 0.05

As identified from the prognostic model, ZC3H12D, N stage and T stage could predict OS of LUAD patients. To explore the relationship between ZC3H12D expression and clinical factors associated with prognosis, we analyzed the significance *P* values by chi-square test. The results showed that T and N stages displayed significant differences between in group C1 and in group C2. In group C1, the expression of ZC3H12D was significantly higher than the median, while in group C2 was significantly lower than the median (Fig. 3a and b). From the correspondent bar chart, it exhibited that the proportion of ZC3H12D high expression in T1 and N0 was significantly higher than that of ZC3H12D low expression (Fig. 3c and d). To further verify the results, we combined N1, N2 and N3 group into one group, which was compared with N0 group to observe the difference of expression between the two groups. In N0 group, the ZC3H12D expression was significantly higher than another group (Wilcoxon rank sum test, *P* = 8.3e-05) (Fig. 3e). In addition, eliminating unknown samples, we took T2, T3 and T4 as a whole. Compared with T1, we found that there was a statistical significance between them and ZC3H1 2D expression was relatively higher in T1 phase (Fig. 3f).

### ScRNA sequencing reveals ZC3H12D expression in normal lung tissue and LUAD

**Fig. 4:**
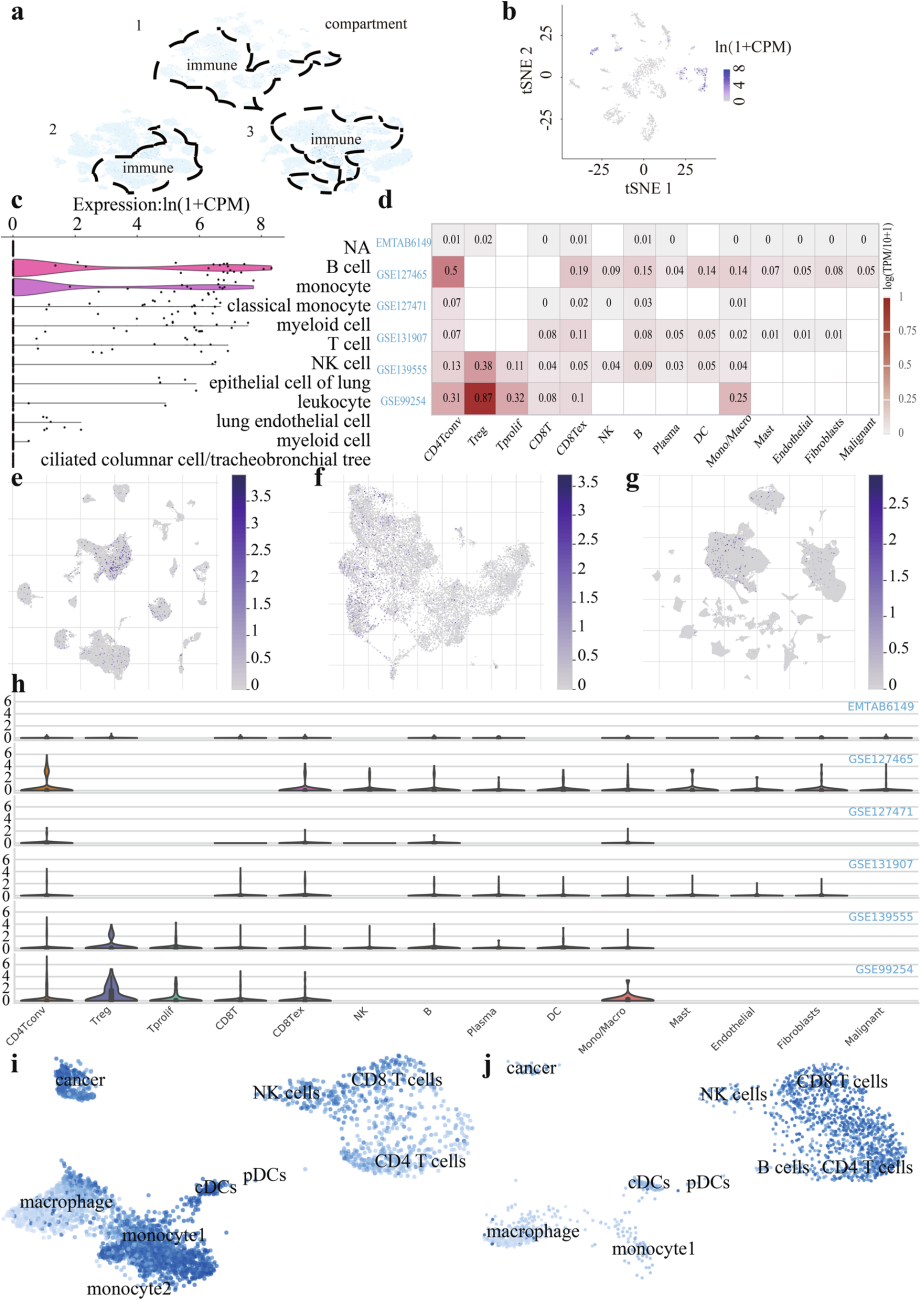
Expression of ZC3H12D in single cell RNA landscapes. **a** The ZC3H12D was found in human lung specimens. **b** The t-SNE analysis of ZC3H12D expression level in mouse lung specimens. **c** Violin diagram of gene expression in different types of cells in mouse lung tissues. **d** Heatmap of ZC3H12D expression displayed heterogeneity in different clusters of cells in different NSCLC datasets. Expression of ZC3H12D in **e** GSE127465, **f** GSE99254 and **g** GSE131907 after umap processing. **h** Violin diagram of gene expression in different immune cells in different NSCLC datasets. **i** The ZC3H12D expression in monocytes was significantly increased in one patient with leptomeningeal metastasis from lung cancer. **j** Expression of ZC3H12D in single-cell RNA sequencing atlas from another patient with leptomeningeal metastasis of lung cancer in the same study

Different from conventional bulk RNA-sequencing, we applied scRNA-seq database to explore the expression level of ZC3H12D in normal lung tissue and LUAD. We found that ZC3H12D was mainly expressed in immune cells, which can be found in both human and mouse lung specimens (Fig. 4a-c). In parallel, we explored data from single-cell RNA sequencing of non-small cell lung cancer in public databases. Using scRNA-seq data from the GEO database, we found that ZC3H12D expression transformed by log (TPM/10 + 1) displayed heterogeneity in different clusters of cells in different NSCLC datasets (Fig. 4d). ZC3H12D was more abundantly expressed in CD4 T cells using GSE127465 and in Treg cells using GSE99254. Then, to eliminate the effect of lung squamous cell carcinoma we used another scRNA-seq dataset from GSE131907 to unravel the expression level in LUAD, indicating that it was relatively higher expressed at CD8 Tex(0.11), CD8 T cells(0.08), B cells(0.08) and CD4 T cells(0.07) over other cells(≤0.05) (Fig. 4e-h). Also, it was expressed in some plasma cells, DC cells, monocytes or macrophages, while ZC3H12D was not abundantly expressed in fibroblasts, mast and endothelial cells. However, when lung cancer metastasized to the brain, the expression levels of various types of cells changed. The ZC3H12D expression in monocytes was significantly increased, even higher than that in CD4 and CD8 T cells sometimes (Fig. 4i). And, the heterogeneity among individuals was also very significant. In another patient with leptomeningeal metastasis of lung cancer, the ZC3H12D expression was relatively higher in CD8 T cells and CD4 T cells (Fig. 4j).

### Screening for important co-expressed genes

**Fig. 5:**
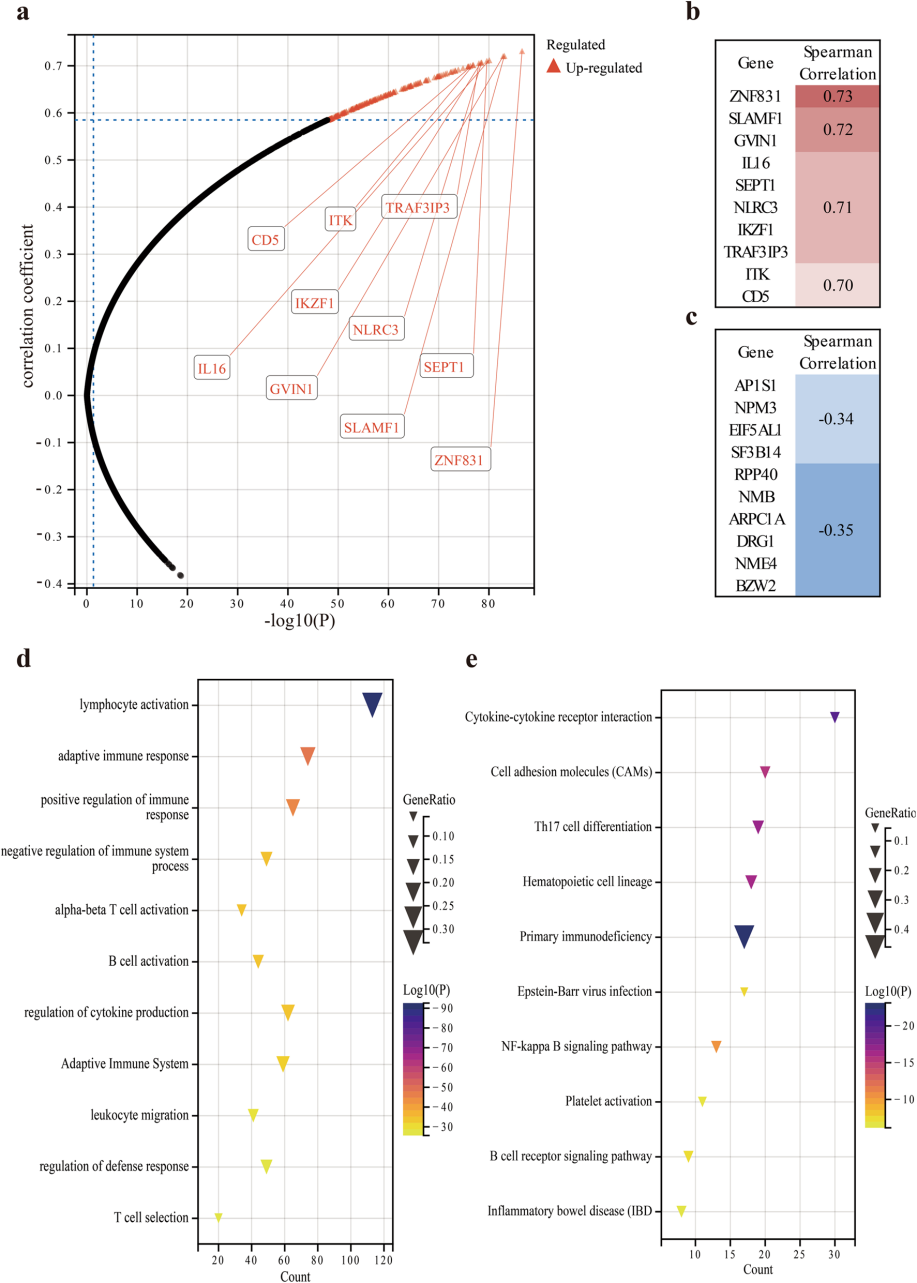
Spearman correlation test. **a** The volcano map showed that a total of 12,201 genes were positively correlated with ZC3H12D expression, while 7786 genes were negatively correlated with ZC3H12D expression. **b** The top 10 positively correlated significant genes and **c** the top 10 negatively correlated significant genes were screened. **d** Bubble map uncovered that genes were mainly enriched in the immune function of lymphocyte activation, immune response, B cell activation and alpha-beta T cell activation. **e** Another bubble chart uncovered the co-expressed genes also participated in many pathways, such as cytokine-cytokine receptor interaction, cell adhesion molecules and Th17 cell differentiation

Base on Spearman correlation test, we identified 19,987 genes from the TCGA LUAD data. A total of 12,201 genes were positively correlated with ZC3H12D expression, while 7786 genes were negatively correlated with ZC3H12D expression, which displayed in the volcanic map (Fig. 5a). The top 10 positively correlated significant genes and the top 10 negatively correlated significant genes were screened and plotted, respectively (Fig. 5b and c).

### GO functional enrichment analysis and KEGG pathway enrichment analysis

After further filtration of 12,201 positive related genes, 345 genes were obtained using correlation coefficient > 0.5, *p*-value < 0.01 and FDR < 0.01 as criteria for screening Gene ontology (GO) classification functional enrichment and Kyoto encyclopedia of genes and genomes (KEGG) classification pathway enrichment of 345 genes were performed. The results demonstrated that these genes were mainly enriched in the immune function of lymphocyte activation, immune response, B cell activation and alpha-beta T cell activation (Fig. 5d and Supplement 3). Meanwhile, co-expressed genes also participated in many pathways, such as cytokine-cytokine receptor interaction, cell adhesion molecules and Th17 cell differentiation (Fig. 5e).

### Correlation between gene expression and immune cell infiltration

**Fig. 6:**
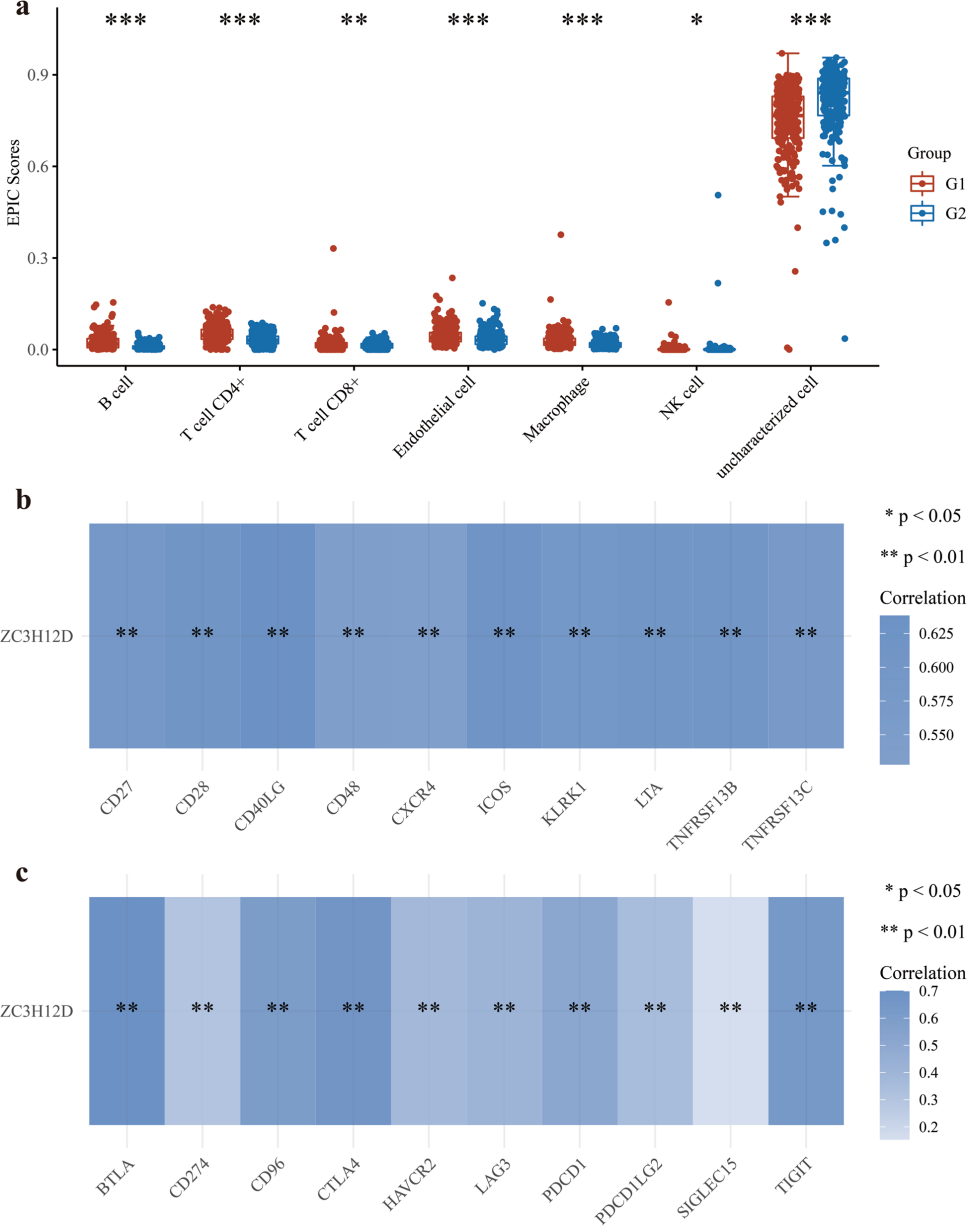
Relationship between ZC3H12D gene expression and immunity. **a** The immune cell score heat map showed the relationship between ZC3H12D gene expression and immune cells. **b** Immunostimulators related gene expression heat map, uncovering the 10 immunostimulators (CD27, CD28, CD40LG, CD48, CXCR4, ICOS, KLRK1, LTA, TNFRSF13B, TNFRSF13C) with statistical significance. **c** The heatmap of the correlation between 10 immunoinhibitors (BTLA, CD274, CD96, CTLA4, HAVCR2, LAG3, PDCD1, PDCD1LG2, TIGIT, SIGLEC15) and ZC3H12D expression. *p < 0.05, **p < 0.01, ***p < 0.001

In the face of so much evidence showing the potential relationship between ZC3H12D and immunity, we decided to explore the correlation between expression of ZC3H12D and various immune cell infiltration levels in LUAD. Divided by the median, G1 group represents high expression of ZC3H12D, while G2 group represents low expression of ZC3H12D. Based on the experimental peritoneal cancer index (EPCI) algorithm, six major immune cells showed a trend: the scores of the G1 group with high expression were higher than the scores of the G2 group with low gene expression, interestingly, in the undefined cells, the scores of the G1 group with high gene expression were relatively lower than those of the G2 group with low gene expression (Fig. 6a).

### Correlation between ZC3H12D and important immune molecules

Five hundred and ten LUAD samples were used for co-expression between ZC3H12D and other genes. Among the co-expressed genes screened, ZC3H12D expression was linked to many immune-related genes. To further explore its regulatory effect, we analyzed the correlation between the expression of ZC3H12D and the common immunostimulators. Ultimately, we identified 10 immunostimulators (CD27, CD28, CD40LG, CD48, CXCR4, ICOS, KLRK1, LTA, TNFRSF13B, TNFRSF13C) with statistical significance (Fig. 6b). Interestingly, we also found that the expression of ZC3H12D was related to immunoinhibitors. 10 statistically significant immunoinhibitors (BTLA, CD274, CD96, CTLA4, HAVCR2, LAG3, PDCD1, PDCD1LG2, TIGIT, SIGLEC15) were also screened, including several immune-checkpoint–relevant transcripts, such as CTLA4, CD96, TIGIT. However, the correlation between the immune checkpoint SIGLEC15 and ZC3H12D expression was relatively weak (Fig. 6c).

## Discussion

Screened from the ceRNA regulatory network of LUAD, ZC3H12D-hsa-miR-4443-ENST00000630242 pathway was found, which was closely related to the survival of patients. High expression of hsa-miR-4443 was not conducive to the prognosis of patients with LUAD, but high expression of ENST00000630242 was beneficial to the prognosis of patients with LUAD. Besides, high ZC3H12D expression was linked to various clinical characteristics and better prognosis. Enriched in immune cells, ZC3H12D was associated with various immune cell infiltration levels and immune molecules. The functional enrichment analysis also showed that the co-expressed genes mainly played a role in lymphocyte activation and cytokine-cytokine receptor interaction.

ZC3H12D is also called MCPIP4, C6orf95, and dJ281H8.1. It is a tumor suppressor gene, which plays a critical role in many cancers, including tongue cancer [52], osteosarcoma [13] and lung cancer. As for lung cancer, most of previous studies focused on the effect of genetic polymorphisms of ZC3H12D, and little was known about its potential regulatory mechanisms on lung cancer [53]. Previous studies have demonstrated that ZC3H12D was associated with memory T lymphocytes and macrophages, participating in the regulation of inflammation [54, 55]. Therefore, we hypothesized that this gene might have an effect on LUAD through immune regulatory mechanism. In our study, we found that many immune-related genes were positively correlated with the expression of ZC3H12D, such as ZNF831, SLAMF1 and IL-16. Both ZNF831 and ZC3H12D were linked to Zinc Finger Family, and it has been reported that ZNF831 was specifically significant in the high immunity subtype of triple-negative breast cancer, which was characterized by anti-tumor immune activities, better immune cell infiltration and greater probability of OS [56]. And SLAMF1 could both inhibit proliferation and impair responses to B cell receptor ligation in IGHV mutated chronic lymphocytic leukemia, which was similar to the anti-tumor effect of ZC3H12D [57]. In the previous literature, ZC3H12D could regulate IL-6, which was a member of the interleukin family, and we further found that IL-16, another member of the interleukin family, was also closely related to ZC3H12D [12, 58]. As a pro-inflammatory cytokine, IL-16 was associated with high grade immune related adverse events in advanced NSCLC treated with immune checkpoint inhibitors [59].

In addition to the correlation of co-expressed genes, it is more convincing to analyze the function and pathway of positively related gene sets in LUAD samples. In our study, a lot of genes participated in the functions and pathways associated with immunity. The function enrichment results showed that many co-expressed genes were involved in lymphocyte activation and immune response, which were consistent with the results of previous studies [60, 61]. In addition to the immune-related pathways, we also found that the co-expressed genes of ZC3H12D expression were enriched in well-known cancer-related pathways, such as NF-kB signaling pathway, which needed to validate its function in cancer in the further.

Besides, the scRNA-seq technology provided us an innovative method to reveal the gene expression level in immune cells under different conditions [62, 63]. Based on the ScRNA-seq in our study, it showed that ZC3H12D was not homogeneous among different clusters in tumor, but it selectively highly expressed in immune cells. Compared with the samples of normal lung tissues, LUAD tissues, non-small cell lung cancer and brain metastasis of lung cancer, we found that the expression of ZC3H12D in different immune cell types, including conventional CD4 T cells, regulatory T cells, monocytes or macrophages, would change under different conditions, which reflected the plasticity of ZC3H12D to a certain extent. Also, when it was highly expressed, the EPIC scores of the main immune cells were correspondingly higher. Meanwhile, the high expression of ZC3H12D was also more common in T1 and N0, and was related to some immune molecules, so we speculated that it may play an anti-cancer effect by regulating immunity in the early stage of LUAD. Future studies are required to compare the immune changes caused by ZC3H12D between early-stage and advanced-stage, so as to further reveal the function of ZC3H12D on the dynamic heterogeneity of LUAD.

Apart from immune mechanism, ceRNA regulatory network may also involve in the prognosis of LUAD. In the present study, lncRNA ENST00000630242, acting as a ceRNA, could “sponge” hsa-miR-4443 to regulate the expression of target ZC3H12D. Overexpression of ZC3H12D was beneficial to prolong the survival time of LUAD patients, while hsa-miR-4443 was not conducive to the prognosis of patients, which were conformity with the pertinent literature [14, 64]. Although there were few studies on ENST00000630242, the data showed that it was beneficial to the prognosis of patients with LUAD. Therefore, ZC3H12D-hsa-miR-4443-ENST00000630242 axis could be served as a novel potential target for LUAD treatment.

The major limitation of this study is that we have not confirmed the ENST00000630242-hsa-miR-4443-ZC3H12D axis by experiments, though the correlations have been primarily uncovered through RNA-seq from clinical samples. Although the function of ZC3H12D was revealed in the present study, the mechanisms of ENST00000630242 and hsa-miR-4443 in LUAD are still unclear. Therefore, further study is needed to explore the role of ENST00000630242 and hsa-miR-4443 in LUAD. Additionally, due to the small number of samples performed by RNA-seq, it might induce bias, so we used the public database to validate our study. In the future, we will also make an effort to enlarge samples to reduce selection bias.

## Conclusion

In summary, we found that ENST00000630242-hsa-miR-4443- ZC3H12D axis might be involved in the OS of LUAD patients. lncRNA ENST00000630242 could “sponge” hsa-miR-4443 to regulate the expression of ZC3H12D. ZC3H12D or ENST00000630242 could be beneficial to improve the prognosis of LUAD patients, while hsa-miR-4443 was not conducive to the overall survival time of patients. ZC3H12D, the core part of this pathway, was combined with some clinical factors to establish a cox model together. Furthermore, ZC3H12D expression at single level was unraveled in both normal lung tissues and lung tumors. Also, we found ZC3H12D expression was associated with some clinical features, important functions and pathways. Meanwhile, we explored the correlation between ZC3H12D and immune mechanisms to understand LUAD better. Therefore, it may serve as a vital predictive marker and could be regarded as a potential therapeutic target for LUAD in the future.

## Supplementary Information

**Additional file 1: Supplementary Figure 1.** a. A ceRNA regulatory network based on 49 mRNAs, 99 miRNAs and 50 lncRNAs. b. Association between ZC3H12D expression and LUAD patient survival time.

**Additional file 2: Supplementary Figure 2.** The survival curves of GNAO1, KSR2, SBK1 and SLIT3.

**Additional file 3: Supplementary Figure 3.** The genes were mainly enriched in the immune function of lymphocyte activation, immune response, B cell activation and alpha-beta T cell activation.

**Additional file 4: Supplementary Table 1.** lncRNA and mRNA ceRNA network list top.

## Data Availability

The datasets generated and analysed during the current study are available, as follows: TCGA database (https://portal.gdc.cancer.gov/), GEO database (https://www.ncbi.nlm.nih.gov/geo/), Tumor Immune Estimation Resource (TIMER, http://timer.cistrome.org/), GO (http://www.bioconductor.org/packages/release/bioc/ html/topGO.html), KEGG (http://www.genome.jp/keggbin/show_organism?menu_type=pathway_maps&org=hsa), miRWalk 2.0 (http://zmf.umm.uni-heidelberg.de/ pps/zmf/mirwalk2/), StarBase (http://starbase.sysu.edu.cn/), KM-plotter (https://kmplot.com/ analysis/), Tabula Muris (https://tabula-muris.ds.czbiohub.org/), European Molecular Biology Laboratory (EMBL, https://www.ebi.ac.uk/), UCSC cell browser (https://cells.ucsc.edu/), TISCH (http://tisch.comp-genomics.org/), LinkedOmics (http://www.linkedomics.org/login.php), Metascape (http://metascape.org/) CNCI (https://github.com/www-bioinfo-org/CNCI), CPC2 (http://cpc2.cbi.pku.edu.cn/), CPAT (https://sourceforge.net/projects/rna-cpat/) and PLEX (https://sourceforge.net/projects/plek/). A dataset generated during the current study is not publicly available due to concerns regarding patient confidentiality and proprietary information but is available upon reasonable request from the corresponding author. We provided a reviewer link of unpublished BioProject. Use the following URL: https://dataview.ncbi.nlm.nih.gov/object/PRJNA732584?reviewer=jo5ph00rjrkr7u19thhft8eho.

## Abbreviations

ZC3H12D: Zinc finger CCCH-type containing 12D
RNA: Ribonucleic acid
ceRNA: Competing endogenous ribonucleic acid
LUAD: Lung adenocarcinoma
OS: Overall survival
CTLA4: Cytotoxic T-Lymphocyte Associated Protein 4
CD: Cluster differentiation
TIGIT: T-cell Immunoreceptor with Ig and ITIM domains
mRNA: Messenger ribonucleic acid
lncRNA: Long non-coding RNA
miRNA: microRNA
NSCLC: Non-small cell lung cancer
CENPF: Centromere proteins F
scRNA-seq: Single-cell RNA sequencing
IER3: Immediate early response 3
TNF: Tumor necrosis factor
IL-6: Interleukin 6
TLR: Toll-like receptors
NF-κB: Nuclear factor kappa-light-chain-enhancer of activated B cells
HCC: Hepatocellular carcinoma
GBM: Glioblastoma
CRC: Colorectal cancer
FAM30A: Family With Sequence Similarity 30 Member A
WHO: World health organization
ncRNA: Non-coding RNA
CPM: Counts per million
CNCI: Coding-non-coding Index
CPAT: Computerized progressive attentional training
CPC2: Coding potential calculator 2
PLEX: Predictor of long non-coding RNAs and mRNAs based on k-mer scheme
DEmRNAs: Differentially expressed mRNAs
DEmiRNAs: Differentially expressed miRNAs
DElncRNAs: Differentially expressed lncRNAs
GO: Gene ontology
MF: Molecular function
BP: Biological processe
CC: Cellular component
KEGG: Kyoto encyclopedia of genes and genomes
TCGA: The cancer genome atlas
EMBL: European molecular biology laboratory
GEO: Gene expression omnibus
RBC: Red blood cell
ROC: Receiver operation characteristic
HR: Hazard ratio
CI: Confidence interval
t-SNE: t-distributed stochastic neighbor embedding
UMAP: Uniform Manifold Approximation and Projection for Dimension Reduction
UCSC: University of California Santa Cruz
TISCH: Tumor immune single-cell hub
FDR: False discovery rate
TIMER: Tumor immune estimation resource
GNAO1: Guanine nucleotide-binding protein G(o) subunit alpha
KSR2: Kachin special region II
SBK1: SH3 domain binding kinase 1
SLIT3: Slit guidance ligand 3
EPCI: Experimental peritoneal cancer index
CXCR4: C-X-C motif chemokine receptor 4
ICOS: Inducible T cell costimulator
KLRK1: Killer cell lectin like receptor K1
LTA: Lymphotoxin alpha
TNFRSF: Tumor necrosis factor receptor superfamily member
MCPIP4: MCP Induced Protein 4
C6orf95: Chromosome 6 open reading frame 95
ZNF831: Zinc finger protein 831
SLAMF1: Signaling lymphocytic activation molecule family member 1
IGHV: Immunoglobulin heavy chain variable region
JAK-STAT: Janus kinase-signal transducer and activator of tran-ions
